# Oviposition strategies in Pieridae butterflies and the role of an egg‐killing plant trait therein

**DOI:** 10.1002/ece3.11697

**Published:** 2024-07-18

**Authors:** Dorette H. Peters, Liana O. Greenberg, Nina E. Fatouros

**Affiliations:** ^1^ Biosystematics Group Wageningen University Wageningen The Netherlands; ^2^ Present address: Laboratory of Entomology Wageningen University Wageningen The Netherlands

**Keywords:** Brassicaceae, evolutionary arms‐races, host plant choice, HR‐like cell death, oviposition preferences

## Abstract

Most herbivorous insects are host‐plant specialists that evolved detoxification mechanisms to overcome their host plant's toxins. In the evolutionary arms‐races between Pieridae butterflies and Brassicaceae plants, some plant species have evolved another defence against the pierids: egg‐killing. Underneath the eggs, leaves develop a so‐called hypersensitive response (HR)‐like cell death. Whether some butterflies have evolved oviposition strategies to counter‐adapt against egg‐killing remains to be studied. In this study, we assessed the oviposition site location of Pieridae butterflies on their natural host plants. We described the plant tissue on which we located the eggs of the most common Pieridae in the Netherlands: *Gonepteryx rhamni, Anthocharis cardamines, Pieris rapae, P. napi, P. brassicae* and *P. mannii*. Additionally, we assessed expression of HR‐like cell death in response to the deposited butterfly eggs. We found that both *A. cardamines* and *G. rhamni* mainly oviposited on the floral stem and the branch, respectively, and oviposited on host plants from lineages not expected to kill pierid eggs. Accordingly, no HR‐like cell death was seen. All *Pieris* eggs found were located on leaves of their host, the only tissue found to express HR‐like cell death. Furthermore, each *Pieris* species was found to at least occasionally oviposit on *Brassica nigra*. This was the only plant species in this survey that expressed HR‐like cell death in response to the eggs of *P. rapae, P. napi* and *P. brassicae*. Our observations demonstrate that HR‐like cell death remains an effective defence strategy against these *Pieris* species and as such did not find evidence for the hypothesized counterstrategies. Surveying certain key species and disentangling the micro‐evolution of oviposition strategies within a species would allow us to further investigate potential counter‐adaptations that evolved against HR‐like cell death. This study provides the basis for further investigation of potential counter‐adaptations to egg‐killing defences.

## Graphical abstract

We screened six Pieridae butterfly species that are commonly found in the Netherlands by documenting their host plant selection, oviposition sites and plant response to oviposition. In agreement with previous screenings in the greenhouse, egg deposition induces leaf necrosis only in certain lineages and tissues. Only *Pieris* species were vulnerable to egg‐killing responses by some of their host plants. These findings suggest that egg‐killing leaf necrosis might have evolved as a novel mechanism to combat specialist herbivores that are adapted to their host plants' toxins.

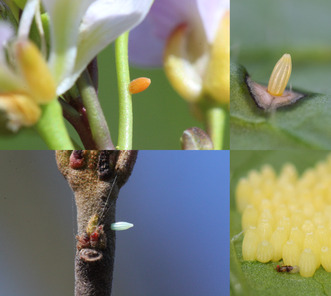

## 1 INTRODUCTION

In the arms‐race between insects and plants, different plant families evolved specific toxins against herbivores (Després et al., 2007). In response, some herbivores evolved mechanisms to detoxify the chemical defences of their host plants (Edger et al., 2015). This results in a coevolutionary arms‐race between the plant and attacker that drives the diversification of plant defence chemicals and, in parallel, drives the evolution of new detoxification mechanisms, host shifts and speciation (Becerra, 2015; Braga et al., 2021; Speed et al., 2015). A well‐studied example of arms‐races between insects and plants are between Pieridae butterflies and Brassicaceae plants. Brassicaceous plants produce secondary metabolites, so‐called glucosinolates, to defend themselves against herbivore attackers (Hopkins et al., 2009). Meanwhile, caterpillars from the Pierinae subfamily have repeatedly evolved gut proteins called nitrile‐specifier proteins (NSP) that allow them to detoxify the evolving glucosinolates of their hosts (Edger et al., 2015; Wheat et al., 2007). While Pierinae larvae are clearly capable of overcoming their hosts' defences, detoxification is only a valuable adaptation if there is an opportunity to feed. An understudied adaptation of plants to escape these specialist herbivores is defence against their egg stage (Griese et al., 2021).

Plants can recognize and respond to herbivore eggs before hatching (Hilker & Fatouros, 2016), which prevents herbivore damage to the plant (Griese et al., 2021). Plant defence mechanisms can cause egg desiccation, dropping, crushing and killing by ovicidal substances (Hilker & Fatouros, 2015, 2016). So far, egg‐killing traits have been documented in more than 30 different plant species across 12 plant families (Fatouros et al., 2016). Until now, research has mainly focussed on toxins against the feeding stages of herbivores and the adaptations developed by these herbivores against them, namely detoxification mechanisms. As a result, interactions between insect eggs and plants have been understudied (Griese et al., 2021).

The killing of herbivorous insect eggs by the development of leaf cell death has been shown for several species within the Brassicaceae and Solanaceae families (Fatouros et al., 2016). Shapiro and DeVay (1987) first described this egg‐killing trait against *Pieris* butterflies in *Brassica nigra*. Eggs laid by *P. rapae* on *B. nigra* were found discoloured, shrivelled and dead due to a necrotic zone in the plant tissue underneath the eggs. This necrotic zone is caused by a hypersensitive response (HR)‐like cell death which resembles HR against pathogens, causing the cytoplasm of cells to become disorganized and granulated followed by cell death. In a later study conducted by Fatouros et al. (2012), induction of HR‐like cell death by *P. brassicae* egg clutches was found in *B. nigra* and some eggs in these clutches did not hatch. Later, Griese et al. (2017) found that gregariously laid *P. brassicae* eggs were less affected by HR. More *P. brassicae* eggs died on necrotic plants when the females were forced to lay single eggs as opposed to their natural oviposition of large egg clutches. Induction of HR‐like cell death by *P. rapae* in *B. nigra* led to egg desiccation and resulted in fewer caterpillars hatching, especially when the cell death responses was very strong (Fatouros et al., 2014). Recent research by Griese et al. (2021) showed that HR‐like cell death was expressed by other Brassicaceous species closely related to *B. nigra* when induced by a wash made of *P. brassicae* eggs. Only plant species in the tribe Brassiceae and a certain *Aethionema* sp. express HR in response to butterfly eggs (Griese et al., 2021). Moreover, only egg wash from different Brassicaceous feeding species of the Pierinae subfamily, including *P. brassicae, P. napi, P. rapae* and additionally *P. mannii* and *Anthocharis cardamines* induced cell death, while wash of non‐brassicaceous feeding Pieridae such as *Gonepteryx rhamni* (subfamily: Coliadinae*), Colias* spp. (subfamily: Coliadinae), *Aporia crataegi* (subfamily: Pierinae) and *Leptidea sinapis* (subfamily: Dismorphinae) induced a lower or no HR‐like cell death (Griese et al., 2021).

Pieridae butterflies may have found ways to overcome mortality due to egg‐killing HR‐like cell death by, for example, clustering their eggs, shifting to other host plants not expressing HR or ovipositing on inflorescences instead of leaves (Griese et al., 2021). Some Pierinae species have been found to oviposit on inflorescences (e.g. *A. cardamines or P. krueperi*) and so far no signs of cell death have been found on inflorescent organs (Griese et al., 2021; van Mastrigt, 2020). Therefore, it seems unlikely that HR‐like symptoms develop on inflorescences. Whether these proposed strategies are used in effect in nature can be investigated by exploring natural patterns of oviposition and the expression of HR in the field.

In this study, we thoroughly assessed the different host plants of Pieridae butterfly species for egg deposition, as well as the responses of these plants to the deposited eggs. To the best of our knowledge, this is the first study that carefully describes the plant part on which eggs were deposited per plant species and cell death response to eggs for Pieridae species. We screened species common to the Netherlands, namely *G. rhamni, A. cardamines, P. rapae, P. napi, P. brassicae* and *P. mannii*. We hypothesize that HR will only be expressed by host plants in the tribe Brassiceae and only in their leaves. Thus, species found to oviposit on other host plants or plant tissues will not induce HR‐like cell death.

## 2 MATERIALS AND METHODS

### 2.1 Plants and insects

Eggs of the following Pieridae species were included in this survey. We also list the host plants that these species are most likely to use in the Netherlands.

*Species of Brassicaceae that are in the lineage with most species that can express HR‐like cell death in response to butterfly eggs (Tribe: Brassiceae) are denoted with a single asterisk (Griese et al., 2021).
**The species that have been previously tested and found to express strong HR‐like cell death in response to butterfly egg wash have been denoted with two asterisks (Griese et al., 2021).

#### 2.1.1 *Gonepteryx rhamni* (Subfamily: Coliadinae, Tribe: Gonepterygini)

Female Brimstone butterflies lay their eggs mainly on juvenile trees of *Rhamnus frangula* (Rhamnaceae). The eggs usually laid not only on the abaxial side of the first fully expanded *R. frangula* leaf within the bud (McKay, 1991), but may also be on a not yet expanded leaf (Eeles, 2019). Occasionally, they may be oviposited near the young leaves on the tip of the branch (Gutierrez & Thomas, 2000) or on the adaxial side of the leaf (Eeles, 2019). Although eggs are laid singly, eggs may be found together as a result of different females laying on the same leaf or a single female returning several times to the same leaf (Eeles, 2019). *Gonepteryx rhamni* egg wash did not induce HR‐like cell death in *B. nigra* (Griese et al., 2021).

#### 2.1.2 *Anthocharis cardamines* (Subfamily: Pierinae, Tribe: Anthocharini)

Females of the orange‐tip butterfly lay their eggs on the inflorescences of larval food plants, mainly wild Brassiceae. Overall, females oviposit single eggs near the siliques or flowers; however, observations of more eggs per plant and on different sites have been made (Agerbirk et al., 2010). The eggs are initially straw‐coloured and turn bright orange after 2–3 days. As the larvae are cannibalistic, normally only one larva completes its development on a single inflorescence (Dempster, 1997). Commonly used host plants include *Alliaria petiolate*, *Cardamine pratensis* and *Arabidopsis thaliana* (Brassicaceae) (Dempster, 1997; Wiklund & Friberg, 2009). Additionally, Sisymbrium officinale, Sinapis arvensis*, Hesperis matronalis, Arabis hirsute, Lunaria annua, C. amara, Brassica rapa* and *Barbarea vulgaris* may be used (Eeles, 2019). Egg wash of *A. cardamines* induced HR‐like cell death in *B. nigra* (Griese et al., 2021).

#### 2.1.3 *Pieris napi* (Subfamily: Pierinae, Tribe: Pierini)

Females of the green‐veined white butterfly lay their eggs on the leaves of several brassicaceous plants in moist habitats, mostly on wild Brassicaceae. They typically lay eggs singly on the abaxial side of a leaf of a small plant that is partially in the shade (Tolman & Lewington, 2009). In the Netherlands, commonly used host plants are Alliaria petiolata, Cardamine pratensis, C. amara, S. arvensis, Raphanus raphanistrum*, Lunaria rediviva, S. officinale, Nasturtium officinale, Brassica oleracea var. oleracea, B. nigra** and Diplotaxis tenuifolia* (Bellmann, 2018; Eeles, 2019; Ellis, 2020a; Heinen et al., 2016; Lewington, 2016). Plants with conspecific eggs are not necessarily avoided (Raitanen et al., 2014). *Pieris napi* (egg wash) is known to induce HR‐like cell death in *B. nigra* (Griese et al., 2021; Shapiro & DeVay, 1987).

#### 2.1.4 *Pieris rapae* (Subfamily: Pierinae, Tribe: Pierini)

Females of the small white lay their eggs on the leaves of various brassicaceous plant species, both on crop and wild plants. The eggs are laid singly on plants and plants with conspecific eggs or larvae are not avoided (Root & Kareiva, 1984). Host plants that are commonly used by *P. rapae* in the Netherlands are: A. petiolata, C. pratensis, B. oleracea**, B. napus**, B. nigra**, S. arvensis*, R. raphanistrum*, Reseda lutea, Tropaeolum majus, S. officinale, Lepidium draba and D. tenuifolia* (Bellmann, 2018; Eeles, 2019; Ellis, 2020b; Heinen et al., 2016; Lewington, 2016). *Pieris rapae* (egg wash) is known to induce HR‐like cell death in *B. montana, B. rapa, B. nigra, H. incana, S. arvensis* and *R. sativus* (Griese et al., 2020, 2021; Shapiro & DeVay, 1987).

#### 2.1.5 *Pieris brassicae* (Subfamily: Pierinae, Tribe: Pierini)

Females of the large cabbage white are gregarious and lay egg clutches containing 40–100 eggs underneath leaves (Lewington, 2016). *Pieris brassicae* uses different host plants based on the season, including both wild and crop plants. In the spring and early summer, they use B. rapa*, during the summer S. arvensis* and in late summer B. nigra** (Fei et al., 2014). Other host plants can be B. oleracea*, B. napus**, R. raphanistrum* and *T. majus* (Bellmann, 2018). Of these host plants, some of their most commonly used are known to express HR‐ like cell death, such as *B. nigra. Pieris brassicae* eggs (or egg wash) is known to induce HR‐like cell death in several species, including *B. montana, B. nigra, Hirschfeldia incana, S. arvensis* and *R. sativus* (Fatouros et al., 2012; Griese et al., 2020, 2021; Pashalidou et al., 2015).

#### 2.1.6 *Pieris mannii* (Subfamily: Pierinae, Tribe: Pierini)

Southern small white females lay single eggs mainly on the underside of leaves from different Brassicaceous host plants. Recently, this species underwent a rapid range expansion. In its native region, the dominant host plants are in the native *Iberis* genus (Neu et al., 2021). *Pieris mannii* populations from more recently colonized areas, such as in neighbouring Germany, were found to have reduced specialization and readily oviposited on a wide range of plants including *Alaria petiolata* and *D. tenuifolia* (Neu et al., 2021). In the Netherlands, eggs are often laid on *Iberis* spp. that are frequently planted as ornamentals in gardens. Apart from *Iberis* plants, in the expanded ranges, eggs are also known to be laid on *Alyssoides utriculatum, L. graminifolium, L. pinnata, C. impatiens, Lobularia maritima* and *Aubrieta deltoidea* (van Mastrigt, 2020). Egg wash of *Pieris mannii* induced HR‐like cell death in *B. nigra* (Griese et al., 2021).

### 2.2 Field survey methods

Fieldwork for this research was conducted in the provinces Gelderland and South Limburg in the Netherlands between April and July 2021 (Table S1). Locations were selected based on anecdotal reports of a high abundance of butterfly and host plant species. Eggs of *G. rhamni* were found at five locations in Renswoude, Wageningen and Wijchen. These locations were near roads and in forests. Eggs of *A. cardamines* were found at 12 locations in Wageningen, Wijchen, Arnhem and Vijlen (South Limburg). All locations were near streams or along paths. All *Pieris* eggs were found along paths, in fields and gardens at 16 locations in Wageningen and Wijchen. All host plants were checked as they were found in the search locations. Therefore, some host plants were found more often than others. For all herbaceous plants, all parts of the plants were checked for eggs. Egg searching on *Rhamnus frangula* is described below. The position of each egg was recorded by the following categories: branch, leaf and flower bud. Additionally, the presence of HR‐like cell death was carefully observed with a magnifier or camera with a macrolens. Cell death was scored as described below. A photograph was taken of almost all found eggs.

### 2.3 Host plant and egg identification

*Rhamnus frangula*, the host plant of *G. rhamni* was identified in the field based on its morphology. All leaves and twigs on branches within a height of ±2 m to the ground were checked for eggs. The eggs were identified by morphology. The trees and eggs were found at five locations (Table S1) between mid‐April and the beginning of June 2021. Eggs laid anywhere on the leaf bud, leaf or petiole were combined into the category ‘leaf’. Other categories were ‘flower bud’ and ‘branch’.

Three main host plants of *A. cardamines*, namely *C. pratensis*, *A. petiolata* and *S. officinale*, were identified in the field from their characteristic morphologies. Eggs of *A. cardamines* were identified by their distinctive morphology and presence of adults at the field site. The plants and eggs were found at 12 locations between end of April until the beginning of June in 2021 (Table S1). Eggs laid directly on the bud were categorized as ‘flower bud’ and eggs on any other part of the inflorescence were categorized as ‘flower stem’. Eggs on stems of siliques were categorized as ‘fruit stem’ and the other categories for these plants were ‘stem’ and ‘leaf’.

As *P. brassicae, P. rapae* and *P. napi* have several overlapping host plants, the following plants were simultaneously checked for all of their eggs: *B. nigra, B. napus, B. rapa, C. pratensis, A. petiolata, B. oleracea, R. raphanistrum, S. officinale, D. tenuifolia* and *S. arvensis*. Using morphological differences between Pieris eggs described by Frohawk (1924), we tried to distinguish *P. rapae* and *P. napi* eggs by counting the number of longitudinal ribs that run along the sides of the eggs from the top to the base (Data S1). Ultimately, this method proved unreliable, and thus, the analysis for all solitary *Pieris* eggs was combined.

*Pieris brassicae* eggs were identified by morphology and their appearance in a clutch. Egg clutches on *B. nigra* were found at two locations in Wageningen from the end of June until the end of July in 2021 and egg development was documented over time.

Similar to *P. rapae* and *P. napi, P. mannii* is a solitary egg laying *Pieris* species. Two host plants were primarily checked for the eggs of *P. mannii*: *Iberis* spp. and *D. tenuifolia*. On both plants, however, occasionally the eggs of the other solitary *Pieris* species could be found. When possible, eggs from these plants were observed for several days to observe the hatching larvae. *Pieris mannii* larvae are distinguishable from *P. napi* and *P. rapae* by their black head capsule. Furthermore, using this feature, several occurrences of *P. mannii* larvae hatching from eggs on other host plants are also reported in the results.

### 2.4 Scoring of HR‐like cell death

Cell death was scored according to Griese et al. (2017), with its severity scored from 1 to 3 and 0 meaning no cell death.

### 2.5 Literature survey methods for phylogenetic character mapping

The Pieridae phylogeny published by Wiemers et al. (2020) was adapted to include only the species that occur in the Netherlands. Mapped traits were as follows: high likelihood of host plant to express HR according to Griese et al. (2020) and (2021), Pieridae eggs induce HR in *Brassica nigra* according to Griese et al. (2021), oviposition on leaves, inflorescence or other plant tissue, and HR observed in the field. For the commonly occurring species we surveyed, characters were mapped using our data from the field. For the remaining species, oviposition traits and natural host plants were retrieved from Tolman (Tolman & Lewington, 2009), Eurobutterflies (http://www.eurobutterflies.com) and UK butterflies (https://www.ukbutterflies.co.uk).

### 2.6 Statistics

Chi‐square tests were performed to examine the relation between the number and position of eggs laid by *G. rhamni* and *A. cardamines*. Chi‐square tests were performed in R (version 2022.12.0) using the R package ‘stats’ (R Core Team, 2022) with function ‘chisq.test’ (Agresti, 2007).

## 3 RESULTS

### 3.1 Oviposition of Coliadinae species on natural host plant

#### 3.1.1 Gonepteryx rhamni

**FIGURE 1:**
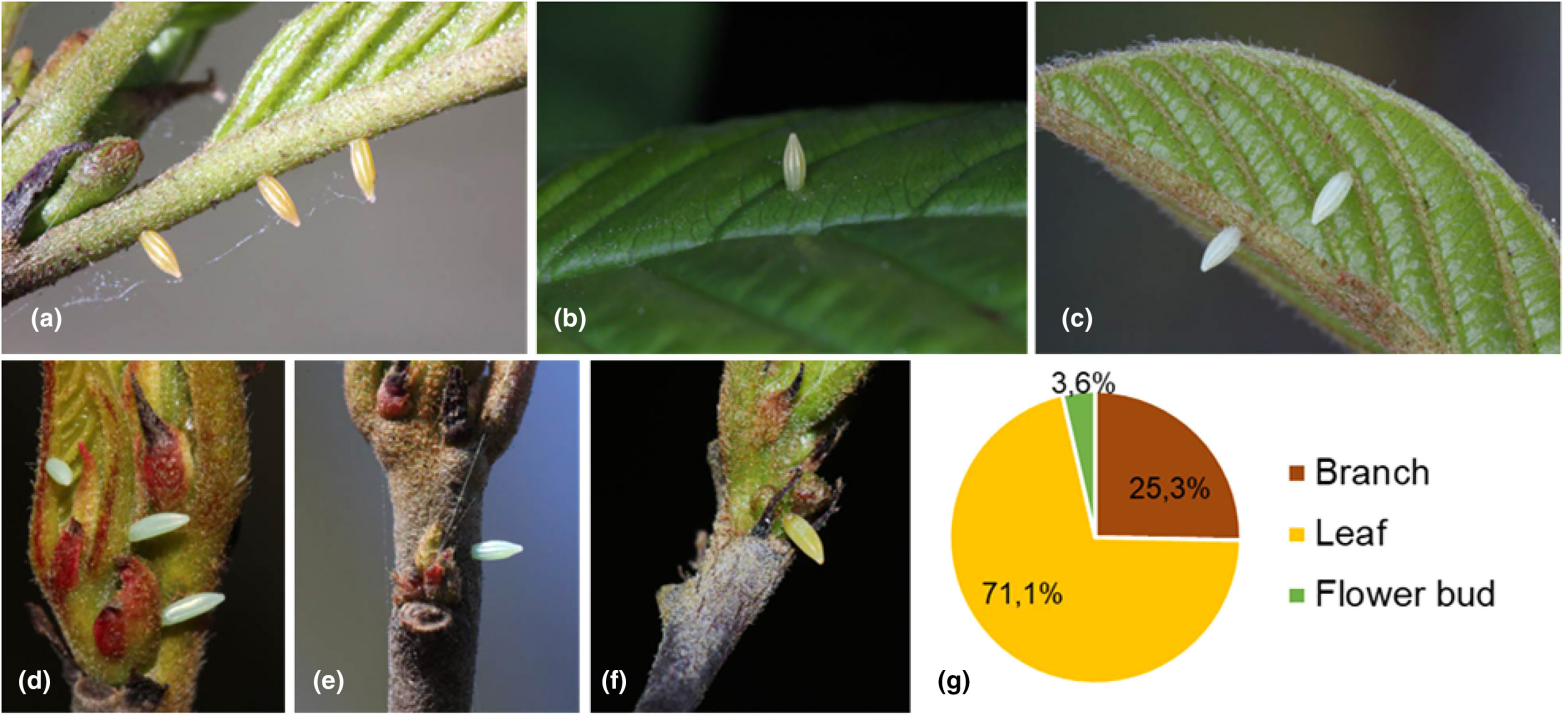
*Gonepteryx rhamni* eggs on *Rhamnus frangula*. (a) petiole and midrib, (b) adaxial side leaf, (c) abaxial side leaf and midrib, (d) leaf bud and midrib, (e) branch, (f) flower bud and (g) pie chart of the percentages of eggs found per position category.

A total of 21 *R. frangula* trees with deposited *G. rhamni* eggs were sampled. In total, 83 eggs were found on these trees. The number of eggs found per position on the trees was significantly different (*Χ* ^2^ (2, *N* = 3) = 59.1, *p* < .001), with the most eggs found on leaves (71.1%), followed by the branches (25.3%) and finally flower buds (3.6%) (Figure 1a–g). No visible HR‐like cell death was observed beneath the eggs monitored on any of the 21 *R. frangula* trees.

### 3.2 Oviposition of Anthocharini (Pierinae) species on natural host plants

#### 3.2.1 Anthocharis cardamines

A total of 272 *A. cardamines* eggs were found on three host plants, showing no induction of HR‐like cell death. Most eggs were found on *C. pratensis* (66.5%), followed by *A. petiolata* (32.5%), and only two eggs were found on *S. officinale* (1%).

**FIGURE 2:**
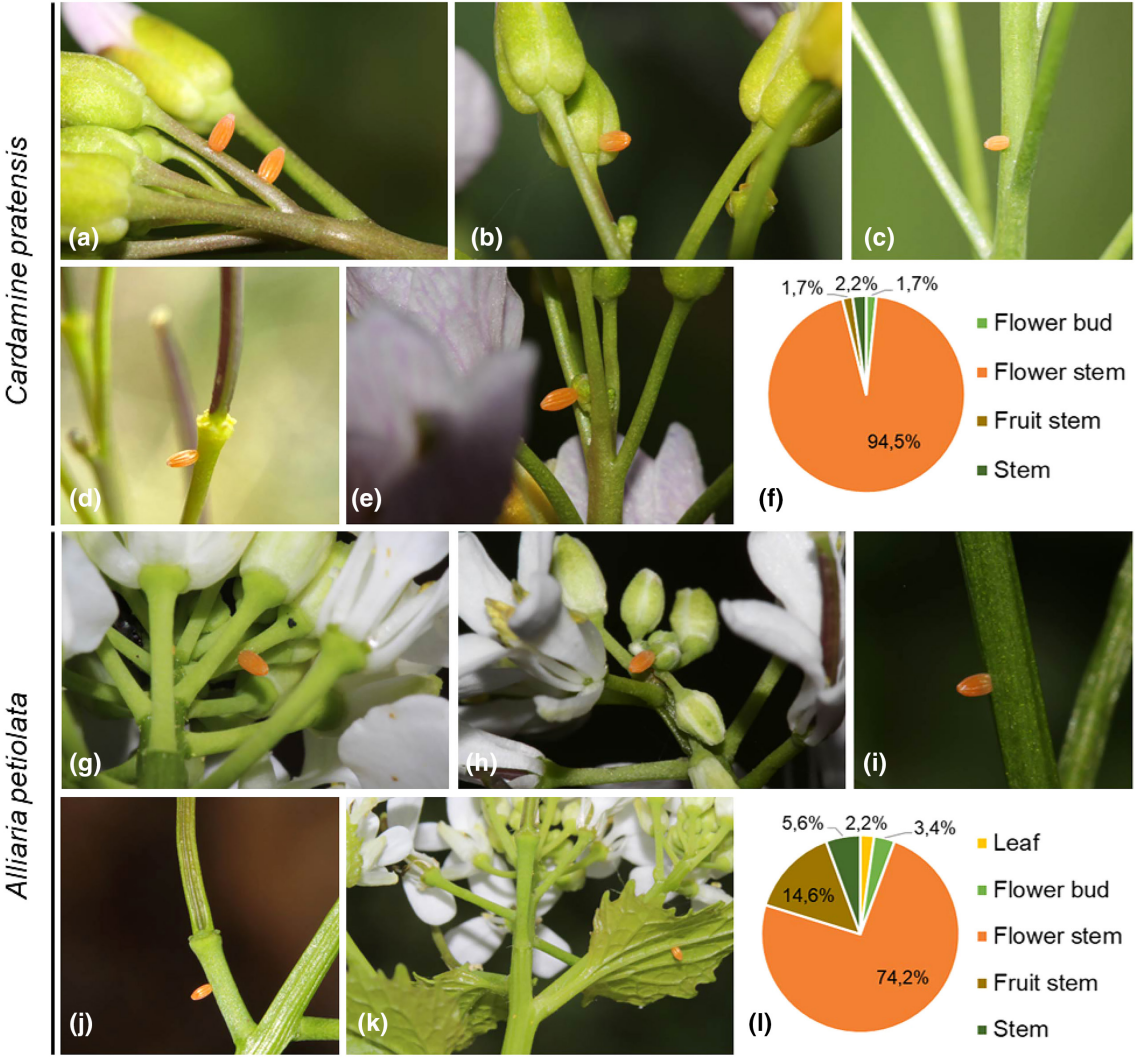
*Anthocharis cardamines* eggs on two host plants. *Cardamine pratensis*: (a) flower stem, (b) flower bud, (c) stem, (d) fruit stem, (e) flower bud and (f) pie chart of the percentage of eggs per position category. *Alliaria petiolata*: (g) flower stem, (h) flower bud, (i) stem, (j) fruit stem, (k) leaf and (l) pie chart of the percentage of eggs per position category.

Significant differences were found for the number of eggs observed on four different positions on *C. pratensis* (*Χ* ^2^ (3, *N* = 4) = 462.98, *p* < .001). Eggs were mainly found on the flower stem (94.5%). Occasionally eggs were found on the fruit stem, stem or flower bud (1.7%, 2.2% and 1.7%, respectively) (Figure 2a–f).

On 93.3% of the examined *A. petiolata* plants, a single egg was found per plant. In addition, a few plants with two eggs were found and one with three or five eggs (3.4%, 1.1% and 1.1%, respectively). There was one exceptional plant with 12 *A. cardamines* eggs (1.1%), and two unidentified white eggs. These 14 eggs were spread over the lateral and terminal inflorescences and shoots. Significant differences were found for the number of eggs observed on five different positions on *A. petiolate* (*Χ* ^2^ (4, *N* = 5) = 167.35, *p* < .0001). Overall, most eggs were found on the flower stem and secondly on the fruit stem (74.2% and 14.6%, respectively). Further eggs were found on the stem, leaf or flower bud (5.6%, 2.2% and 3.4%, respectively) (Figure 2g–l).

On the two *S. officinale* plants examined, each had one egg on the flower stem of a lateral shoot.

### 3.3 Oviposition of different Pierini (Pierinae) species on natural host plants

#### 3.3.1 Pieris brassicae

**FIGURE 3:**
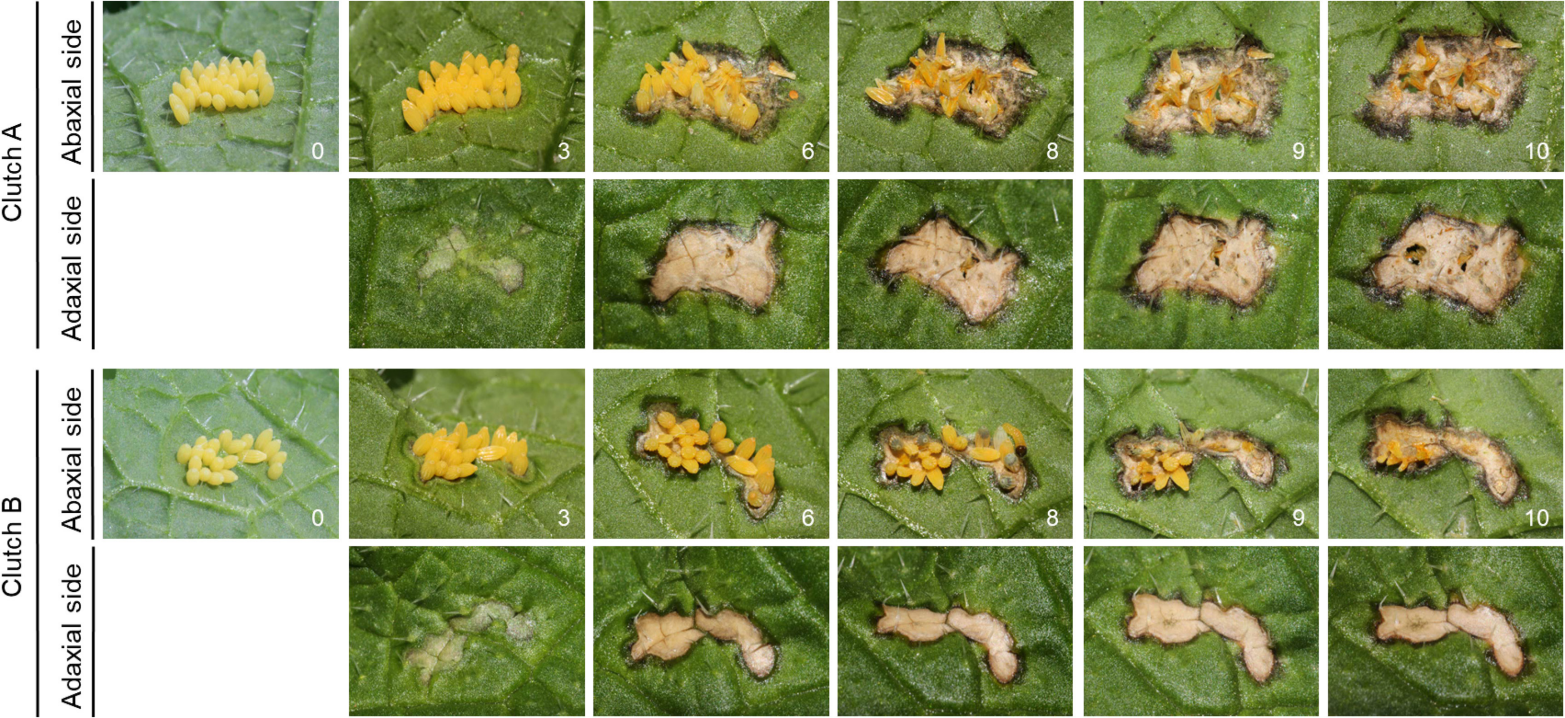
Two *Pieris brassicae* egg clutches on *Brassica nigra*, one with 30 eggs (Clutch A) and one with 20 eggs (Clutch B), both monitored and photographed over a period of 10 days. The upper row shows the abaxial side of the leaf; the lower row shows the adaxial side. The number at the bottom right of photos indicates the number of days after the first observation. Cell death was initially scored as non‐existent (score 0) and eventually scored as severe (score 3).

Six egg clutches of *P. brassicae* were found on two *B. nigra* plants (from now on referred to as clutch A to F), each of which induced varying degrees of cell death. Two of those clutches (A and B) were observed on a single plant over a period of 10 days (Figure 3). Clutch A consisted of 30 eggs (Figure 3a), clutch B of 20 eggs (Figure 3b) and both showed no cell death on the first day they were found (score 0). A severe HR‐like response (score 3) developed underneath both clutches after 6 days. No eggs from clutch A, and from clutch B, only four caterpillars hatched. The other eggs did not survive, probably as a result of HR‐like cell death.

Clutch C consisted of 21 eggs and showed initially no cell death (score 0). After 2 days, the cell death had developed and was scored intermediate (score 2). After 5 days, 14 of the eggs had turned black due to parasitization, the remaining seven shrivelled. Clutch D was found on the same plant as clutch C, containing 45 eggs and showing no cell death (score 0) on the first day. Three of these eggs were almost hatching and one caterpillar had already hatched. After 1 day, cell death developed and was scored with intermediate (score 2). By that time three additional caterpillars hatched, two eggs disappeared and two eggs shrivelled. After 2 days, only seven eggs were left, which were damaged. The other eggs had disappeared.

Clutch E with 79 eggs did not show any cell death (score 0) on the day it was found. After examining the pictures of the clutch, an unidentified species of *Trichogramma* wasp was found parasitizing on the eggs. After 3 days, cell death developed and was scored intermediate (score 2). Four days after discovering the clutch, the cell death was scored as severe (score 3), but all the eggs had disappeared, leaving no remaining eggshells. Clutch F was found on the same plant as clutch E and had 20 eggs that showed no cell death (score 0) on the first day. After 4 days, the cell death was scored with intermediate (score 2). This clutch was not further monitored as the plant was mowed. Clutches E and F were found on the same *B. nigra* plant as clutch A and B, but a month later.

#### 3.3.2 Solitary *Pieris* spp

**FIGURE 4:**
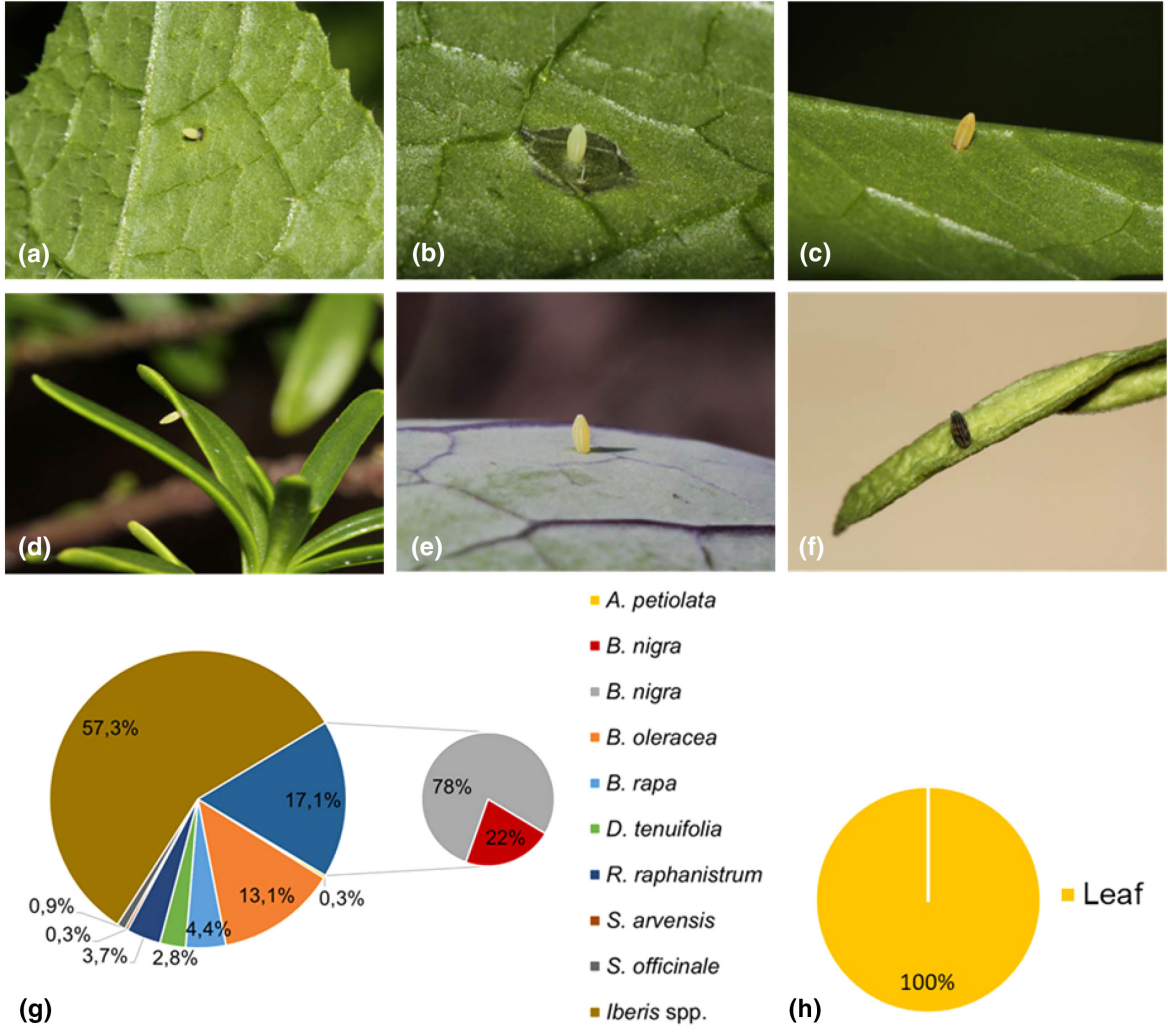
Solitary *Pieris* eggs on different host plants. (a, b) on *Brassica nigra* inducing HR‐like cell death, (c) on *B. nigra* not inducing HR‐like cell death, (d) on *Iberis s*sp., (e) on *B. oleracea*, (f) on *Iberis* sp. and parasitized by a *Trichogramma* sp., (g) pie chart of the percentage of eggs per host plant, for *B. nigra* split into the percentage of plants showing cell death (red) and no cell death (grey) and (h) pie chart of the percentage of eggs per position category.

A total of 321 solitary *Pieris* eggs were found in the field on the following host plants: *A. petiolata* (0.3%), *B. nigra* (17.1%), *B. oleracea* (13.1%), *B. rapa* (4.4%), *D. tenuifolia* (2.8%), *R. raphanistrum* (3.7%), *S. arvensis* (0.3%), *S. officinale* (0.9%) and *Iberis* spp. (57.3%) (Figure 4a–e,g). We could not discriminate singly laid *Pieris* eggs using the method of Frohawk (1924) as the number of ribs was overlapping between *P. napi* and *P. rapae* eggs (Data S1). The eggs were all found on various parts of the leaves (Figure 4a‐f). Twelve eggs found on *B. nigra* induced cell death in the leaves with a score varying from mild to severe (score 1–3), thus 22% of solitary *Pieris* eggs on *B. nigra* induced cell death (Figure 4a,b,g). Of those, two eggs, whose cell death was scored intermediate (score 2) and severe (score 3) were missing after a few days, but no caterpillar was found. Out of one egg (cell death scored with a 2), a caterpillar hatched. Other eggs were not monitored over time. All other eggs found on *B. nigra* did not induce HR‐like cell death and neither did eggs found on all other host plants.

Nearly all eggs found on *Iberis* spp. (Figure 4d) that could be identified as larvae were found to be *P. mannii* (>90%, personal observation). This accounted for most of *the P. mannii* observed in this study; however, several *P. mannii* eggs were found on a *Diplotaxis* sp., and notably, also on *B. nigra* on two occasions. No eggs on *Iberis* spp. or *Diplotaxis* sp. were found to have induced HR and of the 12 solitary eggs that did induce HR on *B. nigra*, none could be confirmed as *P. mannii* or otherwise.

### 3.4 Mapping host plant usage, the presence of oviposition and host plant traits on Pieridae phylogeny

**FIGURE 5:**
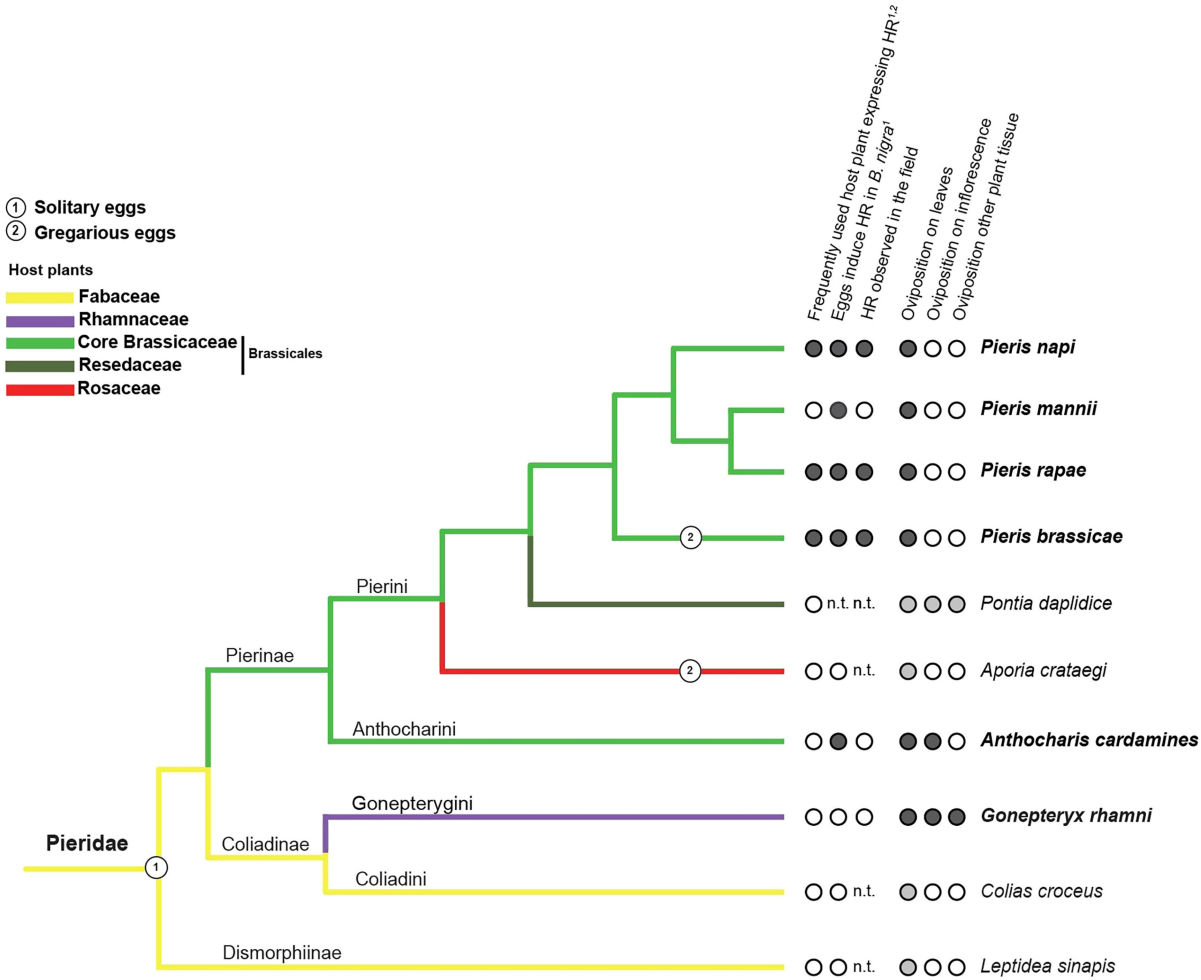
Mapping host plant usage, the presence of oviposition and host plant traits on a recently published Pieridae phylogeny by Wiemers et al. (2020). We focused only on species that can be found in the Netherlands, and those which occur commonly and were screened in this study are written in bold (Lewington, 2016). Filled dark circles denote presence observed in this study, empty circles absence according to this study and/or literature and filled grey circles presence according to the literature. n.t. not tested. The branches in the Pieridae phylogeny are coloured to primary host plant associations of the various lineages: Fabaceae (yellow), Rhamnaceae (purple), core Brassicaceae (bright green) and Resedaceae (dark green)—the latter two belonging to the Brassicales, and lastly Rosaceae (red). Mapped traits were as follows: High likelihood of host plant to express HR according to Griese et al. (2020)^1^ and Griese et al. (2021)^2^, Pieridae eggs induce HR in *Brassica nigra* according to Griese et al. (2021)^1^, oviposition on leaves, inflorescence or other plant tissue and HR observed in the field. Oviposition traits and natural host plants were retrieved from Tolman and Lewington (2009), Eurobutterflies (http://www.eurobutterflies.com) and UK butterflies (https://www.ukbutterflies.co.uk). Most Pieridae species are solitary and lay single eggs (1) with a few exceptions namely *Pieris brassicae and Aporia crataegi* which are gregarious species and lay clustered eggs (2).

The literature‐based expectations and field‐based observations for the species studied in this system, as well as the theoretical expectations for the other most commonly found Pieridae species in the Netherlands, are reported below (Figure 5). *Gonepteryx rhamni* eggs are expected to be found on Rhamnaceae hosts, and as such, should not and did not induce HR in the field. *Anthocharis cardamine* eggs can be found on a range of brassicaceous hosts; however, the dominantly used species are not likely to express HR and the dominantly used plant tissues, flowers and stems, are also not expected to express HR. As such, in our study, the species found with *A. cardamine* eggs were *C. pratensis* and *A. petiolata* and no occurrences of HR expression were documented. *Pieris brassicae* eggs were found on *B. nigra*, a host plant known to express strong HR‐like cell death in response to *Pieris* eggs. All six clutches found indeed had strong HR expression. *Pieris rapae* and *P. napi* eggs could not be distinguished, but both were found on the leaves of the expected range of host plants. Only on *B. nigra*, known to express HR in response to solitary *Pieris* eggs, did we observe HR‐like cell death. *Pieris mannii* is expected to use *Iberis* plants, which do not express HR. Most *P. mannii* were found on *Iberis* and indeed no HR was expressed. They are also known to occasionally oviposit on inflorescence, which was not seen in our survey. Additionally, as known for their expanded range, *P. mannii* eggs were found on alternate host plants, including *D. tenuifolia* and *B. nigra*. We have no known instances of *B. nigra* expressing HR in response to *P. mannii* eggs. On *B. nigra*, the vast majority of eggs were either *P. rapae*, *P. napi* or *P. brassicae*, and thus, it is not expected that any of the 12 eggs that did induce HR were likely to be *P. mannii*. Of the species we did not survey, neither eggs from *Leptidea sinapis* (Dismorphiinae) nor *Colias croceus* (Coliadinae) induce HR in *B. nigra* or their natural host plant. Wash made from *Aporia crataegi* eggs did not or only slightly induce cell death in *B. nigra* (Figure 5). Whether eggs of the Western bath white *Pontia daplidice*, which lays its eggs on inflorescences, induce HR in their host plants remains to be studied.

## 4 DISCUSSION

In this field survey, we found 891 Pieridae eggs and for each egg recorded the host plant and plant tissue on which it was laid, as well as whether the plant showed HR‐like cell death underneath the egg. Neither eggs of *G. rhamni* nor *A. cardamines* induce HR‐like cell death in their natural host plants, and their eggs were mainly laid on plant parts other than the leaves. For *Pieris* species, eggs were always laid on the leaves and cell death was only observed on *B. nigra* leaves but not on *A. petiolate*, *B. oleracea*, *B. rapa, D. tenuifolia*, *R. raphanistrum*, *S. arvensis*, *S. officinale*, nor *Iberis* spp. Cell death was never observed on other plant tissues than leaves.

*Gonepteryx rhamni* belongs to the Coliadinae subfamily of the Pieridae, which does not feed on Brassicaceae. Unlike the other Fabaceae feeding Coliadinae, the Gonepteryx butterflies use Rhamnaceae as host plants (McKay, 1991) (Figure 1). In this study, all eggs of *G. rhamni* were found on *R. frangula*. To the best of our knowledge, no Rhamnaceae are known to express HR‐like cell death in response to any insect eggs (Griese et al., 2021). Eggs were mainly laid on the branches, young leaves and yet unfolded leaves as earlier described by McKay (1991) and Gutierrez and Thomas (2000). Multiple eggs were found on the same leaves and branches, most likely due to different females laying on the same leaf or a single female returning (Eeles, 2019). As expected, no cell death was found in any of the tissue types where eggs were laid, including leaf tissues.

Of the Pierinae subfamily, which all feed on Brassicaceae, we surveyed *A. cardamines* and four species of *Pieris* namely: *P. brassicae, P. rapae, P. napi* and *P. mannii*. Of these, two were not found to induce HR‐like cell death in their brassicaceous host plants:

*Anthocharis cardamines*: Species in the Anthocharini tribe also feed on brassicaceous plants; however, their NSPs evolved separately from *Pieris* (Edger et al., 2015) and their preferred host plants are also slightly shifted from the other Pierinae in this study (Tribe: Pierini). Eggs of *A. cardamines* were mainly laid on the flower stems of *C. pratensis*, *A. petiolata* and *S. officinale*, and occasionally, eggs were found on the fruit stem, stem, flower bud or leaves, which corresponds with previous findings (Agerbirk et al., 2010; Dempster, 1997; Wiklund & Åhrberg, 1978). The main host plant used in our study, *C. pratensis*, has very small leaves that were never found to have eggs. On hosts with bigger leaves, such as *A. petiolata*, *A. cardamine* eggs were occasionally found on leaves, but never found to induce HR‐like cell death. While egg wash of *A. cardamines* induced HR‐like cell death in the leaves of *B. nigra* (Griese et al., 2021), it is not surprising that eggs did not induce a necrotic response in the leaves of *A. petiolata* nor *C. pratensis*, as both belong to clades where expression of HR‐like cell death is not known (Griese et al., 2021). Thus, the fact that most *A. cardamines* eggs were laid on tissues that do not express HR cannot be attributed in these cases to a counterstrategy to avoid HR responses, as none would be expected. Nonetheless, the potential hosts of *A. cardamines* do include some plants that have the potential to express HR, and on these plants, *A. cardamines* propensity to oviposit on tissues that should be refuges from HR may still be a benefit.

*Pieris mannii*: The solitary *Pieris* eggs found on *Iberis* plants are likely to be laid by *P. mannii*; however, it cannot be excluded that some of these eggs were *P. rapae*. Nonetheless, HR‐like cell death was never observed on *Iberis* sp. plants in this survey, and therefore, it can be concluded that *P. mannii* does not induce HR‐like on *Iberis* spp. However, previously we have shown that egg wash made from *P. mannii* eggs induced a strong HR‐like cell death and high pathogenesis‐related (PR1) gene expression in *B. nigra* (Griese et al., 2021). Thus, *P. mannii* eggs are likely to contain the elicitor inducing cell death. The chemical nature of this elicitor is currently under investigation (Caarls et al., 2023). One species belonging to the genus *Iberis* has so far been tested (*I. amara*) and likewise does not show HR‐like cell death (Griese et al., 2021). *Iberis* species belong to lineage II outside the Brassiceae. Unlike the Brassiceae, tribe, this lineage did not go through a genome triplication event which is hypothesized to have enabled plants to evolve an HR‐like cell death (Griese et al., 2021). Together with the findings in this study, it seems unlikely that any *Iberis* spp. shows HR‐like cell death. This lack of egg‐killing defence could have been a benefit for *P. mannii*, leading to specialization on this genus as opposed to the use of other brassicaceous hosts with egg‐killing responses used by its sister species *P. rapae*. However, the species *P. mannii* is rapidly expanding its range throughout Europe. It has been hypothesized that this is due to an expansion in host plant use, so *P. mannii* is no longer exclusively found on *Iberis* plants (Neu et al., 2021). Our observations support this switch, as we have seen *P. mannii* laying eggs on *D. tenuifolia* and *B. nigra*. Interestingly, a *P. mannii* female was observed bending her abdomen towards a flower bud of *I. umbellifera* as if she wanted to lay an egg. The actual eggs were not noticed; however, an egg was previously found on a flower bud of a *Diplotaxis* spp. (van Mastrigt, 2020). If *P. mannii* is shifting to plants that do express HR, alternate strategies such as oviposition on flowers may be selected for. Also of note was the presence of several parasitized eggs on *Iberis* plants, that are presumed to be *P. mannii* eggs. *Trichogramma* spp. emerged from approximately 10% of these eggs, an association which to the best of our knowledge has not yet been reported.

Of the remaining solitary *Pieris* eggs, eggs were found on *A. petiolata, B. nigra, B. oleracea, B. rapa, D. tenuifolia, R. raphanistrum, S. arvensis* and *S. officinale* host plants. *Pieris rapae* and *P. napi* eggs could not be identified to species level, as they were morphologically indistinguishable. Additionally, it cannot be excluded that *P. mannii* had also laid eggs on these alternate hosts, as discussed above. Moreover, host plants cannot be used to identify which butterfly species laid the eggs as we have seen both *P. mannii* and *P. rapae* hatching from eggs laid on the same host plant. Therefore, all solitary *Pieris* eggs on these plants were grouped for analysis. All eggs were laid on leaves, as expected for these species. Some eggs induced cell death, but only those found on *B. nigra*. The HR‐like cell death showed to be an effective defence mechanism in some cases, but one caterpillar hatched from the egg, nevertheless. No clear strategies to overcome HR‐like cell death (e.g. ovipositing on the inflorescence) were identified. Interestingly, eggs from the Noctuidae family were found on six *B. nigra* plants and never induced a necrotic response. This confirms previous observations made for *Mamestra brassicae* (also from the Noctuidae family) which neither induced any HR response in *B. nigra* (Caarls et al., 2023; Fatouros et al., 2012; Griese et al., 2021). Three times, the *Pieris* eggs on the same plant induced a cell death response, even when the noctuid eggs did not. This suggests that the cell death response in *B. nigra* is specific to *Pieris* eggs. HR‐like cell death was not found in *B. oleracea*, which was not expected as Griese et al. (2021) found enhanced expression in 20%–40% of their plants. However, in this study, eggs were found on nine plants, of which seven were found at the same farm and were, therefore, most likely laid on the same genotype. It could be that the *B. oleracea* plants found in this study were not of a genotype that expresses HR‐like cell death. HR‐like cell death was not expressed for any of the other host plants in this study.

All *P. brassicae* egg clutches were found on *B. nigra*. HR‐like cell death was induced by all six clutches and was an effective defence against three clutches where most of the eggs did not hatch. Interestingly, the research by Griese et al. (2017) showed that HR‐like cell death was not as effective due to slowed‐down desiccation caused by the eggs being laid in a clutch. It has been suggested that clustering of eggs may increase parasitism and predation, and in this study, both egg parasitism and HR‐like cell death for the same egg clutch were observed, suggesting a double defence mechanism. Previously, it was found that eggs inducing HR‐like cell death in *B. nigra* showed higher parasitism rates than eggs that did not induce cell death (Fatouros et al., 2014). One *P. brassicae* egg clutch was parasitized within 5 days after the first observation, and on another clutch, a parasitoid wasp was found (*Trichogramma* sp.) while parasitizing the eggs. The latter egg clutch was found missing after 4 days, just as a part of another egg clutch. These ‘missing’ eggs were most likely predated upon by other arthropods. Thus, we found that *P. brassicae* egg clutches were highly vulnerable to both HR and egg parasitism in our study.

The induction of HR in Pieridae butterflies evolved in the Pierinae tribe in concordance with the colonization of the Brassicaceae and the appearance of glucosinolates detoxification (Edger et al., 2015). It appears that only eggs of Pierinae species feeding on Brassicaceae elicit cell death on the leaves of plants in the Brassiceae tribe (Griese et al., 2021). Exceptions within the Pierinae include the Black‐veined white *A. crataegi*, which stopped using Brassicales some 37 Ma years ago (Edger et al., 2015), and egg wash does not or only slightly induce cell death in *B. nigra* (Griese et al., 2021). So far, *A. crataegi* eggs have not been observed to induce HR in its natural host plants belonging to the Rosaceae (Nina E. Fatouros, personals observations). Certain key species remain unsurveyed, such as *Pontia daplidice* whose natural host plant species *Reseda luteola* belongs to the Resedaceae (Tolman & Lewington, 2009), a family within the Brassicales that is not expected to respond with HR (Edger et al., 2015). Another key species to survey which does not occur in the Netherlands is *Pieris ergane*, which lays solitary eggs on host plants of the distantly related *Aethionema* genus. Other than *P. mannii*, which specialized on *Iberis* spp. until recently, *P. ergane* is the only *Pieris* to be a specialist on plants outside of the tribe Brassiceae. As one *Aethionema* species was previously found to express HR‐like necrosis to *Pieris* egg wash (Griese et al., 2021), it is also one of the only Brassicaceous plants that is distantly related to the tribe Brassiceae known to do so. Whether eggs of the *P. ergane* or *P. daplidice* induce HR in their host plants in nature remains to be studied.

Furthermore, it is possible that micro‐evolution of elicitors, oviposition strategies and receptor genes in butterflies and plants is occurring at the population level. For example, we have observed certain alpine populations of the rapidly evolving species *P. napi* with a propensity to lay eggs on flowers. It would be highly informative to investigate these differences in oviposition tissue selection on the population level to assess whether these populations laying on flowers avoid egg‐killing responses more successfully than the leaf‐laying population we studied. It remains unknown whether the butterflies can assess their host plant's likelihood of expressing egg‐killing HR. Behavioural choice assays using genotypes of plants that do or do not express HR will allow us to investigate this detection ability of the butterflies. However, regardless of the butterflies' ability to directly assess the chemical differences signalling the likelihood egg‐killing, natural variation in butterflies' egg‐laying strategies allows for the selection for counteradaptations. If many *Pieris* eggs do not survive on certain host plant tissues or lineages with high egg‐killing, host shifts could eventually occur due to the survival advantage of butterfly genotypes favouring oviposition on safer plant tissues and species.

Overall, our field observations and literature search support that HR‐like cell death in response to butterfly eggs evolved specifically against the Pierinae and in the Brassiceae plants they use most often. Specifically, we found that the genus *Pieris* remained at risk of egg‐killing by their host plants, where oviposition on flowers is not utilized as a counter‐adaptation in the specific population we studied. Also unique to *Pieris* was their exclusive deposition of eggs on leaves. It is interesting to consider why this genus is so likely to lay eggs on leaves when leaves are the only tissue that can effectively kill their eggs. This is especially interesting considering that species such as *P. brassicae* are known florivores, eventually moving to the flowers to feed (Lucas‐Barbosa et al., 2014), but still oviposit exclusively on leaves. One explanation could be that, compared to the other species in our study that use more visually apparent hosts, *Pieris* use less apparent plants and therefore rely on the use of gustatory chemical cues while assessing sites for oviposition, as seen by frequent alighting on non‐host plant leaves (Wiklund, 1984). Thus, the chemical cues in the leaves seem particularly important to guide *Pieris* egg deposition compared to the other species in our study, which could explain their propensity for oviposition on leaves. This is also evidenced by the tarsal gustatory receptors of adult *P. brassicae* that detect glucosinolates in their hosts' leaves (van Loon et al., 1992). Therefore, it could be that only in these plants that are most subjected to the leaf‐based oviposition by the *Pieris* butterflies (e.g. the Brassiceae tribe and certain *Aethionema* species) that selection pressure would be consistently strong enough for the plants to evolve a mechanism to kill eggs on leaves. In other words, for example, *Anthocharis* butterflies distribute their eggs across multiple plant tissues, including flowers and stems where cell death does not occur. Therefore, the benefit of cell death would be greatly reduced in their hosts and their hosts may not face the consistent strong selection pressure necessary to drive the evolution of egg‐killing in the arms‐race between Pieridae and Brassicaceae.

## 5 CONCLUSION

In the arms‐race between brassicaceous plants and pieridae butterflies, several counter‐adaptations to egg‐killing HR‐like cell death have been suggested. We provide a first attempt to show evidence of this hypothesis in the field. Based on our observations, there is no evidence that pieridae butterflies try to avoid HR‐like cell death by switching to other host plants, ovipositing on plant tissues that do not respond to eggs with HR‐like cell death, or laying egg clutches that are more resistant to the consequences of cell death in egg mortality. Our survey of Pieridae in the Netherlands revealed that the species vulnerable to egg‐killing, *Pieris* spp., are most likely to lay eggs on tissues and plants that would express HR, namely on leaves of *B. nigra*. We have seen several cases of successful egg‐killing by plant cell death, including egg clutches. It seems most likely that the arms‐race between eggs from Pierinae species and Brassicaceous plants is still ongoing. Future studies should collect similar data from an increased number of butterfly and plant species in the field and survey natural variation in oviposition choices between butterfly populations, for example, including *P. napi* populations with a propensity to lay eggs on flowers. With such data, it would be possible to trace these characters onto a phylogeny with increased resolution and to further understand the evolution of egg‐killing HR in the Brassicaceae and counteradaptations in the Pieridae.

## AUTHOR CONTRIBUTIONS

**Dorette H. Peters:** Conceptualization (equal); data curation (lead); formal analysis (lead); funding acquisition (equal); methodology (lead); writing – original draft (lead); writing – review and editing (equal). **Liana O. Greenberg:** Conceptualization (equal); formal analysis (supporting); funding acquisition (equal); methodology (equal); supervision (equal); writing – original draft (supporting); writing – review and editing (equal). **Nina E. Fatouros:** Conceptualization (equal); funding acquisition (lead); methodology (supporting); project administration (lead); supervision (equal); writing – review and editing (equal).

## CONFLICT OF INTEREST STATEMENT

The authors declare no conflict of interest.

## Supporting information


Data S1.

Table S1.

## Data Availability

The data that support the findings of this study are openly available in DRYAD, https://datadryad.org/stash/share/utqGtJtGZS7DP2bxj6ZCW1P5jl3aPkjTw6sjH9qZpHQ.
