# Supplementary material for: Oviposition strategies in Pieridae butterflies and the role of an egg‐killing plant trait therein
Source: Ecol Evol. 2024 Jul 18;14(7):e11697. doi: 10.1002/ece3.11697 (PMC11257707; doi:10.1002/ece3.11697)
Supplement: Supplementary file 1 — Data S1. [file ECE3-14-e11697-s001.docx]

**Attempts at morphological egg identification**

A morphological identification method was tested to see if *Pieris napi* and *P. rapae* eggs are distinguishable. Eggs from both species look very much alike as they are similar in size and colour. This newly used method was based on the number of longitudinal ribs that run along the sides of the eggs from the top to the base. Frohawk (1924) describes that *P. napi* has fourteen or fifteen ribs, of which nine or ten run from top to bottom, and the other five start a little under the top and run to base. For *P. rapae* there are twelve ribs, of which ten run from top to bottom, and two start a little under the top and branch from the other ribs (Frohawk, 1924). Based on this information it was expected that eggs with twelve ribs or less would be *P. rapae* and eggs with thirteen to fifteen ribs would be *P. napi*. To be able to count the ribs of the eggs, pictures were taken with a phone and magnifier of the top of the egg so all ribs would be pointing out. The ribs were then carefully counted. However, it was found that this method could not be used reliably. There were numerous cases where an egg was laid by a known species, and when the number of ribs on the egg was subsequently checked, it did not match expectations. Therefore, we found that the number of ribs on *P. napi* and *P. rapae* eggs considerably overlapped using our counting method such that this method could not be used to distinguish species. The number of ribs on *P. mannii* eggs was not checked. Therefore, all solitary *Pieris* eggs were grouped for analysis.
