# Supplementary material for: Oviposition strategies in Pieridae butterflies and the role of an egg‐killing plant trait therein
Source: Ecol Evol. 2024 Jul 18;14(7):e11697. doi: 10.1002/ece3.11697 (PMC11257707; doi:10.1002/ece3.11697)
Supplement: Supplementary file 2 — Table S1. [file ECE3-14-e11697-s002.docx]

Table S1: Locations of host plants and eggs per butterfly species.

| *Gonepteryx rhamni* |  |  |  |  |
| --- | --- | --- | --- | --- |
| **Location** | **Coordinates** | **Host plant species** | **Number of trees** | **Number of eggs** |
| De Oude Tol, Wageningen | 51°59'12.7"N 5°40'47.6"E | *R. frangula* | 8 | 35 |
| Hernense bos, Hernen | 51°49'43.7"N 5°41'39.7"E | *R. frangula* | 3 | 6 |
| Kavelpad, Wijchen | 51°49'08.7"N 5°43'25.4"E | *R. frangula* | 1 | 5 |
| Randweg, Wijchen | 51°49'08.0"N 5°43'33.0"E | *R. frangula* | 7 | 32 |
| Wittenoordseweg, Renswoude | 52°04'47.6"N 5°31'32.9"E | *R. frangula* | 2 | 5 |
| **Total** |  |  | **21** | **83** |
|  |  |  |  |  |
| *Anthocharis cardamines* |  |  |  |  |
| **Location** | **Coordinates** | **Host plant species** | **Number of plants** | **Number of eggs** |
| Van Cittersweg, Balgoij | 51°47'34.8"N 5°42'15.1"E | *C. pratensis* | 112 | 148 |
| Akkermaalsbos, Wageningen | 51°58'54.3"N 5°39'23.8"E | *C. pratensis* | 28 | 32 |
| Veerdam, Wageningen | 51°57'45.9"N 5°41'16.2"E | *A. petiolata* | 6 | 6 |
| De Blauwe Bergen, Wageningen | 51°58'55.6"N 5°39'46.3"E | *A. petiolata* | 1 | 1 |
| Dijkgraaf, Wageningen  Bornsesteeg, Wageningen  Bakenbergseweg, Arnhem  Zwanensingel, Wijchen  Geertjesweg, Wageningen  Dolderstraat, Wageningen  Zoomweg, Wageningen  Cottessen, Vijlen | 51°58'48.6"N 5°39'22.6"E  51°59'07.4"N 5°39'42.1"E  52°00'38.4"N 5°52'19.6"E  51°49'02.6"N 5°43'52.1"E  51°58'30.6"N 5°41'16.7"E  51°58'36.7"N 5°41'20.2"E  51°58'49.7"N 5°41'27.4"E  50°45'50.8"N 5°56'38.7"E | *A. petiolata*  *A. petiolata*  *A. petiolata*  *A. petiolata*  *A. petiolata*  *A. petiolata*  *S. officinale*  *C. pratensis* | 14  1  40  3  3  1  2  1 | 16  1  56  3  5  1  2  1 |
| **Total** |  |  | **212** | **272** |
|  |  |  |  |  |
| *Solitary Pieris eggs* |  |  |  |  |
| **Location** | **Coordinates** | **Host plant species** | **Number of plants** | **Number of eggs** |
| Honingblokpad, Wageningen  WUR Student Farm, Wageningen  WUR Student Farm, Wageningen  WUR Student Farm, Wageningen  Unilever, Wageningen | 51°58'47.2"N 5°41'07.2"E  51°58'53.9"N 5°39'26.1"E  51°58'53.9"N 5°39'26.1"E  51°58'53.9"N 5°39'26.1"E  51°59'03.6"N 5°40'00.2"E | *S. officinale*  *B. oleracea*  *D. tenuifolia*  *R. raphanistrum*  *B. nigra* | 2  7  4  1  11 | 2  11  4  12  17 |
| Veenhof, Wijchen  Radix, Wageningen  Radix, Wageningen  Unifarm, Wageningen  Orion, Wageningen  Aalsburg, Wijchen  Aalsburg, Wijchen  Uiterwaarden, Wageningen  Campus Plaza, Wageningen  De Blauwe Bergen, Wageningen | 51°49'11.0"N 5°44'15.7"E  51°59'11.2"N 5°39'50.5"E  51°59'11.2"N 5°39'50.5"E  51°59'15.6"N 5°39'47.2"E  51°59'04.7"N 5°39'56.9"E  51°48'31.3"N 5°42'43.8"E  51°48'31.3"N 5°42'43.8"E  51°57'51.3"N 5°40'59.1"E  51°58'57.4"N 5°39'50.9"E  51°58'55.6"N 5°39'46.3"E | *S. arvensis*  *B. nigra*  *S. officinale*  *B. nigra*  *B. nigra*  *B. oleracea*  *B. rapa*  *B. nigra*  *B. oleracea*  *A. petiolata* | 1  19  1  1  1  1  1  1  1  1 | 1  31  1  1  1  22  14  5  9  1 |
| Saltshof, Wijchen  Veenhof, Wijchen  Veerweg, Wageningen  Ritzema bosweg, Wageningen  Diedenweg, Wageningen  Mennonietenweg, Wageningen  Rijnveste, Wageningen  Leeuweriksweide, Wageningen  Veerweg, Wageningen | 51°49'26.4"N 5°43'47.2"E  51°49'10.9"N 5°44'14.3"E  51°57'52.9"N 5°40'43.0"E  51°58'12.4"N 5°40'32.5"E  51°58'09.3"N 5°41'02.1"E  51°57'59.8"N 5°39'18.9"E  51°58'41.1"N 5°38'56.8"E  51°58'31.3"N 5°39'17.7"E  51°57'52.9"N 5°40'43.0"E | *Iberis* sp.  *Iberis* sp.  *Iberis* sp.  *Iberis* sp.  *Iberis* sp.  *Iberis* sp.  *Iberis* sp.  *Iberis s*p.  *D. tenuifolia* | 1  1  n.a.  n.a.  n.a.  n.a.  n.a.  3  n.a. | 4  2  113  20  9  14  19  3  5 |
| **Total** |  |  | **55** | **321** |
| *Pieris brassicae* |  |  |  |  |
| **Location** | **Coordinates** | **Host plant species** | **Number of plants** | **Number of eggs** |
| Radix, Wageningen  Unifarm, Wageningen | 51°59'11.2"N 5°39'50.5"E  51°59'15.6"N 5°39'47.2"E | *B. nigra*  *B. nigra* | 1  1 | 149  66 |
| **Total** |  |  | **2** | **215** |

n.a. not available
